# Comparison of penh, fluka, and Geant4/topas for absorbed dose calculations in air cavities representing ionization chambers in high‐energy photon and proton beams

**DOI:** 10.1002/mp.13737

**Published:** 2019-08-19

**Authors:** Kilian‐Simon Baumann, Felix Horst, Klemens Zink, Carles Gomà

**Affiliations:** ^1^ Department of Radiotherapy and Radiooncology University Medical Center Giessen‐Marburg Marburg Germany; ^2^ Institute of Medical Physics and Radiation Protection University of Applied Sciences Giessen Germany; ^3^ GSI Helmholtzzentrum für Schwerionenforschung Darmstadt Germany; ^4^ Frankfurt Institute for Advanced Studies (FIAS) Frankfurt Germany; ^5^ Department of Oncology, Laboratory of Experimental Radiotherapy KU Leuven Leuven Belgium

**Keywords:** beam quality correction factors, dosimetry, high‐energy photon and proton radiation, Monte Carlo simulation, radiation therapy

## Abstract

**Purpose:**

The purpose of this work is to analyze whether the Monte Carlo codes penh, fluka, and geant4/topas are suitable to calculate absorbed doses and $f_{Q}/f_{Q_{0}}$ ratios in therapeutic high‐energy photon and proton beams.

**Methods:**

We used penh, fluka, geant4/topas, and egsnrc to calculate the absorbed dose to water in a reference water cavity and the absorbed dose to air in two air cavities representative of a plane‐parallel and a cylindrical ionization chamber in a 1.25 MeV photon beam and a 150 MeV proton beam — egsnrc was only used for the photon beam calculations. The physics and transport settings in each code were adjusted to simulate the particle transport as detailed as reasonably possible. From these absorbed doses, $f_{Q_{0}}$ factors, $f_{Q}$ factors, and $f_{Q}/f_{Q_{0}}$ ratios (which are the basis of Monte Carlo calculated beam quality correction factors $k_{Q,Q_{0}}$) were calculated and compared between the codes. Additionally, we calculated the spectra of primary particles and secondary electrons in the reference water cavity, as well as the integrated depth–dose curve of 150 MeV protons in water.

**Results:**

The absorbed doses agreed within 1.4% or better between the individual codes for both the photon and proton simulations. The $f_{Q_{0}}$ and $f_{Q}$ factors agreed within 0.5% or better for the individual codes for both beam qualities. The resulting $f_{Q}/f_{Q_{0}}$ ratios for 150 MeV protons agreed within 0.7% or better. For the 1.25 MeV photon beam, the spectra of photons and secondary electrons agreed almost perfectly. For the 150 MeV proton simulation, we observed differences in the spectra of secondary protons whereas the spectra of primary protons and low‐energy delta electrons also agreed almost perfectly. The first 2 mm of the entrance channel of the 150 MeV proton Bragg curve agreed almost perfectly while for greater depths, the differences in the integrated dose were up to 1.5%.

**Conclusion:**

penh, fluka, and geant4/topas are capable of calculating beam quality correction factors in proton beams. The differences in the $f_{Q_{0}}$ and $f_{Q}$ factors between the codes are 0.5% at maximum. The differences in the $f_{Q}/f_{Q_{0}}$ ratios are 0.7% at maximum.

## 1 Introduction

Current national and international dosimetry protocols for the determination of absorbed dose in photon beams (e.g., AAPM TG‐51^1^ or IAEA TRS‐398^2^ or the DIN 6800‐2^3^) as well as proton beams (e.g., IAEA TRS‐398^2^) are based on standards of absorbed dose to water. The absorbed dose to water can be determined with air‐filled ionization chambers. When using these chambers, the user needs to correct the chamber reading with the beam quality correction factor $k_{Q,Q_{0}}$. This correction factor accounts for the different response of the chamber in the calibration beam quality $Q_{0}$ and the clinical or user beam quality *Q* (e.g., MV photons or high‐energy protons) and typically corrects the ionization chamber reading by a few percent. Ideally, these $k_{Q,Q_{0}}$ factors should be determined directly using calorimetry for each chamber model used and at exactly the radiation quality *Q* at which the chamber will be operated. Although performed in several studies,^4^, ^5^, ^6^, ^7^, ^8^ the experimental determination of these $k_{Q,Q_{0}}$ factors requires a high experimental effort and is not convenient for most laboratories. Furthermore, standard laboratories do not have access to all beam qualities *Q* — this holds especially for proton and carbon ion beams. Hence, the calculation of beam quality correction factors by means of Monte Carlo simulations is an efficient alternative.

At the time, the IAEA TRS‐398 Code of Practice (CoP) is currently being updated. Within this framework, the RTNORM project^9^ is supporting the IAEA working group with experimental as well as Monte Carlo calculated $k_{Q,Q_{0}}$ factors for different ionization chambers and beam qualities such as photons and protons. Whereas the use of Monte Carlo codes for the determination of $k_{Q,Q_{0}}$ factors in high‐energy photon and electron beams has been extensively tested and is well established in the literature for the Monte Carlo codes egsnrc and penelope,^10^, ^11^, ^12^, ^13^, ^14^ data for protons are scarce with only one study by Gomà et al.^15^ where penh was used to calculate $k_{Q,Q_{0}}$ factors in clinical proton beams in agreement with experimental data within 1% or better. Furthermore, data for ions heavier than protons are nonexistent. Although penh has been shown appropriate for the calculation of $k_{Q,Q_{0}}$ factors in proton beams,^15^ this code cannot transport ions heavier than protons. Hence, to provide more data for protons and especially heavier ions, the use of general purpose codes such as fluka and Geant4, primarily designed for high‐energy physics applications, needs to be investigated for the use in ionization chamber calculations. A first study proved that TOPAS, a toolkit based on Geant4, may be used to calculate $f_{Q}$ factors in proton beams.^16^ On this basis, its usage for the determination of beam quality correction factors in clinical photon and especially proton beams shall be investigated in this work.

Hence, the aim of this work is to assess whether fluka and geant4/topas are suitable to calculate $k_{Q,Q_{0}}$ factors in clinical photon and proton beams. To do so, the $f_{Q_{0}}$ and $f_{Q}$ factors as well as the $f_{Q}/f_{Q_{0}}$ ratios were calculated for simplified beam settings and simplified geometries representing ionization chambers at typical water depths. The $f_{Q}/f_{Q_{0}}$ ratio is the basis of $k_{Q,Q_{0}}$ factors and the only part that can be calculated with the Monte Carlo method. The residual part consists of the $W_{\mathrm{air},Q}$/$W_{\mathrm{air},Q_{0}}$ ratio that has to be determined experimentally (or can be taken from the literature^17^). Hence, if a Monte Carlo code is able to calculate $f_{Q}/f_{Q_{0}}$ ratios, it can be used to determine $k_{Q,Q_{0}}$ factors. We compared the results with the already established Monte Carlo codes egsnrc and penh. The comparison of the $f_{Q_{0}}$ factors with the results from egsnrc is necessary to verify if fluka and geant4/topas can be used for photon calculations since egsnrc is well established for high‐energy photon calculations and benchmarked against experimental data.^18^, ^19^ The comparison of the $f_{Q}$ factors and $f_{Q}/f_{Q_{0}}$ ratios with the results from penh is necessary to verify if fluka and geant4/topas can be used for the calculation of $f_{Q}/f_{Q_{0}}$ ratios and hence $k_{Q,Q_{0}}$ factors in proton beams since penh has been shown appropriate for the calculation of $k_{Q,Q_{0}}$ factors in proton beams.^15^ By choosing simplified beam settings and simplified geometries, we could ensure the use of the same geometry descriptions in each of the Monte Carlo codes used so that only the differences in the particle transport and the physics settings will have an impact on the absorbed dose predictions. The particle transport parameters and the lists of physics models used in each of these Monte Carlo codes were adjusted to simulate the transport of primary photons and protons as well as their secondary electrons at clinically relevant energies as detailed as reasonably possible.

## 2 Materials and methods

Simulations were performed with the Monte Carlo codes penh, fluka, geant4/topas, and egsnrc. In the next subsection, the geometry and beam parameters are explained. In the following subsection, the calculated quantities are described. In the subsections thereafter, the particle transport for each of the Monte Carlo codes used is described. To report the Monte Carlo simulations, we followed the recommendations of the AAPM Research Committee Task Group 268.^20^

### 2.A. Geometries, materials, and source parameters

**Figure 1:**
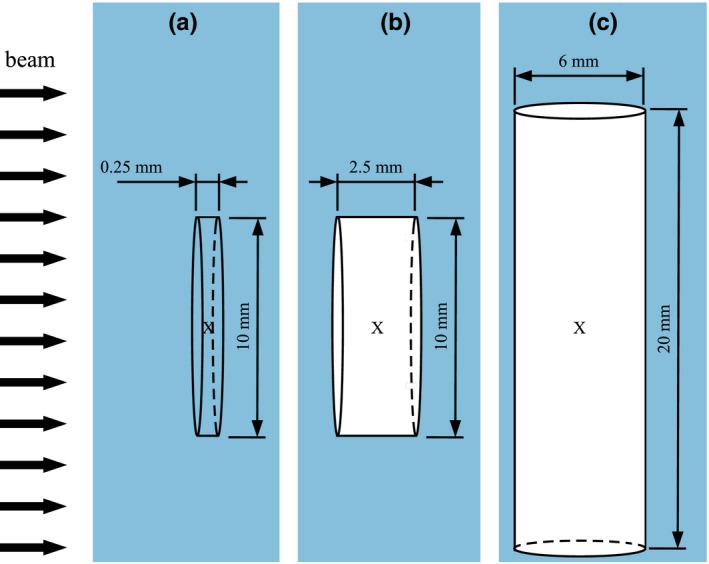
Geometries used for the simulations: (a) reference volume: a water‐filled plane‐parallel volume with a diameter of 10 mm and a height of 0.25 mm, (b) air‐filled plane‐parallel volume with a diameter of 10 mm and a height of 2.5 mm and (c) air‐filled cylindrical volume with a height of 20 mm and a diameter of 6 mm. The direction of the broad beam is marked with black arrows on the left. [Color figure can be viewed at http://www.wileyonlinelibrary.com/]

For each Monte Carlo code, the geometries as shown in Fig. 1 were used: A water cavity used as reference consisting of a disk with a diameter of 10 mm and a thickness in beam direction of 0.25 mm. The first air cavity was a disk with a diameter of 10 mm and a thickness in beam direction of 2.5 mm representing a plane‐parallel chamber. The investigated volume was ∼0.2 cm${}^{3}$, comparable to the Scanditronix NACP02 chamber with an active volume of 0.16 cm${}^{3}$ or the PTW‐34001 Roos chamber with an active volume of 0.35 cm${}^{3}$.^21^

The second air cavity was a cylinder with a height of 20 mm and a diameter of 6 mm representing a cylindrical ionization chamber. The investigated volume was ∼0.6 cm${}^{3}$, comparable to the Farmer chamber Exradin A12 with an active volume of 0.65 cm${}^{3}$ 22 or the NE2571 chamber with an active volume of 0.69 cm${}^{3}$.^21^

These volumes were positioned in a water phantom of 20 × 20 × 15 cm${}^{3}$ for the irradiation with photons and 20 × 20 × 10 cm${}^{3}$ for protons. The center of each volume marked with an “**x**” in Fig. 1 was positioned in the center of the beam at a depth of 5 cm in the water phantom for the irradiation with photons and at a depth of 2 cm for protons.

Table 1 shows the elemental compositions of water and air used in the Monte Carlo simulations. For all elements, only the main isotopes were considered (e.g., no ${}^{17}$O but only ${}^{16}$O in water as well as air). Water had a density of 1.0 g/cm${}^{3}$ and a mean ionization potential of $I_{w}\;=\;78$ eV.^17^, ^23^ For air, the density was 1.20479 mg/cm${}^{3}$ and the mean ionization potential was set to $I_{\mathrm{air}}\;=\;85.7$ eV.^17^

**Table 1:** Elemental compositions of water and air used in the simulations. All fractions are given in mass fractions.

Element	Water	Air
${}^{1}$H	0.111894	0.0
${}^{12}$C	0.0	0.000124
${}^{14}$N	0.0	0.755268
${}^{16}$O	0.888106	0.231781
${}^{40}$Ar	0.0	0.012827

As a ${}^{60}$Co source, we used a monoenergetic 1.25 MeV photon beam^14^ applied in an homogeneous field of 10 × 10 cm${}^{2}$. The beam had no divergence and the space between the source and the water phantom was filled with vacuum.

For the irradiation with protons, the same settings were used. The energy of the protons was 150 MeV (also monoenergetic). The simulations for protons were not performed with the egsnrc code since it can only transport photons, electrons, and positrons.

Concerning the beam quality and depths of the volumes, the setup corresponds to the recommendations of the TRS‐398 Code of Practice for the 1.25 MeV photons.^2^ For monoenergetic protons, TRS‐398 suggests the plateau region at a depth of 3 g cm${}^{-2}$. However, several works^24^, ^25^ established a depth of 2 g cm${}^{-2}$ for protons, which is the depth also used in this work.

### 2.B. Calculated quantities

The absorbed dose to water ($D_{w}$) in the water‐filled volume from Fig. 1 and the absorbed dose to air ($D_{\mathrm{air}}$) in the air‐filled volumes were calculated with each Monte Carlo code for photons and each code excluding egsnrc for protons.

From these results, the factor $f_{Q}$ was calculated as^12^:

(1) $$f_{Q}={\left(\frac{D_{w}}{D_{\mathrm{air}}}\right)}_{Q}$$

where *Q* denotes the beam quality.

Using this factor $f_{Q}$, the beam quality correction factor $k_{Q,Q_{0}}$ can be derived^26^:

(2) $$k_{Q,Q_{0}}=\frac{f_{Q}}{f_{Q_{0}}}\frac{W_{\mathrm{air},Q}}{W_{\mathrm{air},Q_{0}}}$$

where $W_{\mathrm{air}}$ is the mean energy required to create an ion pair in air for the beam qualities $Q_{0}$ and *Q*. In this work, the beam quality $Q_{0}$ corresponds to 1.25 MeV monoenergetic photons applied in a homogeneous, parallel beam of 10 × 10 cm${}^{2}$ representing a ${}^{60}$Co beam and the beam quality *Q* corresponds to 150 MeV monoenergetic protons applied in a homogeneous, parallel beam of 10 × 10 cm${}^{2}$.

In this study, only the ratios $f_{Q}/f_{Q_{0}}$ from Eq. (2) and not the beam quality correction factors $k_{Q,Q_{0}}$ were investigated since the $f_{Q}/f_{Q_{0}}$ ratios are the only part of the beam quality correction factors that can be calculated with Monte Carlo codes. The ratio of the $W_{\mathrm{air}}$ values has to be determined experimentally or can be taken from the literature (e.g., the ICRU report 90^17^). Additionally, the aim of this study is to investigate whether the Monte Carlo codes used are feasible for ionization chamber calculations in general and not to calculate any real beam quality correction factors.

In addition to the determination of the $f_{Q}/f_{Q_{0}}$ ratios, the spectra of the primary particles (photons or protons) as well as the spectra of secondary electrons were scored in the water‐filled reference volume in order to try to explain differences in the particle transport and physics models for the different Monte Carlo codes.

As shown in a recent study by Pfuhl et al.,^27^ the dose buildup effects which are present in the entrance channel of a proton Bragg curve provide an excellent test of both the electromagnetic interaction models (delta electron buildup in the first few millimeters) as well as the nuclear interaction models (secondary proton buildup in the first few cm) implemented in a radiation transport code. Therefore, the integrated depth–dose curve of 150 MeV protons was calculated with all three studied proton transport codes (penh, fluka, geant4/topas). The Bragg curve was scored in a 10 × 10 × 20 cm${}^{3}$ water phantom when irradiating with a pencil beam of 150 MeV protons. The same physics settings as in the cavity simulations were used. The binning of the scored dose distribution was 10 × 10 cm${}^{2}$ laterally and 0.1 mm in the direction of beam.

In the following subsections, the Monte Carlo codes used are described shortly.

### 2.C. Monte Carlo code 1: penh

penh 28 is an extension of the Monte Carlo code penelope 29 that includes the transport of protons based on their electromagnetic interactions in matter. Proton nuclear interactions and prompt‐gamma emission are included for a limited number of isotopes: ${}^{1}$H, ${}^{12}$C, ${}^{14}$N, ${}^{16}$O, ${}^{31}$P, ${}^{40}$Ca.${}^{30}$ Both penelope and  penh have been reported to pass the Fano test within 0.1%^31^, ^33^ for the energy range of interest to this work. Furthermore, penh has been shown to yield $k_{Q}$ factors in proton beams in good agreement with experimental data.^15^ Photon, electron, and positron cross sections, as well as transport simulation parameters are described in detail in Ref. [29] Cross sections for proton electromagnetic interactions are described in Ref. [28] Cross sections for proton nuclear interactions and prompt‐gamma emission are described in Ref. [30], as it is also described the approximate transport of secondary charged particles heavier than protons. Neutrons are not transported.

To improve efficiency without compromising on accuracy, the geometry of all penh simulations was constructed as follows^15^: (a) a scoring volume (see Fig. 1), (b) a 540 μm‐thick “skin” (around the scoring volume) — with a thickness equal to the *continuous slowing down approximation* range ($R_{\mathrm{CSDA}}$) in water of a 200 keV electron, multiplied by a safety factor of 1.2 to account for the possibility that an electron may travel a distance beyond its $R_{\mathrm{CSDA}}$ due to energy‐loss straggling^31^ (c) a 5 mm envelope around the skin and (d) the water phantom. In the scoring volume and 540 μm‐thick skin, we performed detailed simulation (i.e., every single interaction was simulated as a catastrophic event^32^); whereas in the 5 mm‐thick envelope and the water phantom, we used a mixed (class II^32^) simulation scheme. The absorption energies ($E_{\mathrm{abs}}$) and transport simulation parameters ($C_{1}$, $C_{2}$, $W_{\mathrm{cc}}$, $W_{\mathrm{cr}}$ and dsmax) used in these regions are detailed in Table 2. No variance reduction techniques were used.

**Table 2:** Absorption energies and transport simulation parameters used in penh simulations.

Region	$E_{\mathrm{abs}}\left(e^{-}\right)$	$E_{\mathrm{abs}}\left(\gamma \right)$	$E_{\mathrm{abs}}\left(e^{+}\right)$	$E_{\mathrm{abs}}\left(p\right)$	$C_{1}$	$C_{2}$	$W_{\mathrm{cc}}$	$W_{\mathrm{cr}}$	dsmax
Scoring volume	1 keV	1 keV	1 keV	1 MeV	0	0	0	0	n/a
540 μ*m* skin	1 keV	1 keV	1 keV	1 MeV	0	0	0	0	n/a
5 mm envelope	200 keV	1 keV	200 keV	1 MeV	0.05	0.05	10 keV	1 keV	200 μm
Water phantom	200 keV	1 keV	200 keV	1 MeV	0.1	0.1	10 keV	1 keV	2 mm

As the main program, we used peneasy.^34^ We scored the energy deposited in the scoring volume with the tallyEnergyDeposition and the fluence differential in energy with the tallyFluenceTrackLength. The output of the tallyEnergyDeposition (in units of eV/history) was converted to absorbed dose (in units of gray) by converting eV to joules (J) and dividing the energy by the density and volume of the scoring volume. The output of the tallyFluenceTrackLength (in units of cm/eV per history) was converted to ${\text{MeV}}^{-1}\;\cdot \;{\text{cm}}^{-2}$ by converting eV to MeV and dividing by the volume of the water cavity. Statistical uncertainties were estimated using the history‐by‐history method.^29^

### 2.D. Monte Carlo code 2: fluka

The second code chosen for the comparison is the Monte Carlo code fluka 35, 36 (FLUKA2011 Version 2c.6). Originally developed for high‐energy physics applications, nowadays it is also widely used for simulations in proton and heavy ion therapy.^37^, ^38^, ^39^, ^40^ The code is capable of transporting various kinds of particles including photons, electrons, positrons, neutrons, protons, and heavy ions. Charged particles can be transported down to 1 keV and their energy loss is treated in a condensed history approach. Single Coulomb scattering events are condensed in a multiple scattering algorithm. Hadron–nucleus interactions are treated via the PEANUT model. The models implemented in fluka are under ongoing development^38^ and are frequently updated.

Also for fluka, a Fano cavity test was performed by Lourenco et al.^41^ (for proton transport only) with the result that fluka passes the test within 0.15% if the step size in the multiple Coulomb scattering algorithm is set small enough compared to the dimensions of the cavity of interest (step size of 0.01 cm in the case of Lourenco et al. where the radii of the plane‐parallel cavities were between 0.78 cm and 4.08 cm and the cavities were positioned at a depth where the charged particle equilibrium is reached^41^).

In fluka, the user can in most cases not choose between different physics models (unlike, e.g., in Geant4), but only enhance their precision level in certain steps. On the one hand, this reduces the flexibility of the code but, on the other hand, its predictions are well reproducible and very robust.

For all fluka simulations performed in this work, the physics models were set to the highest precision level (e.g., full Rayleigh and Coulomb scatter corrections, heavy fragment evaporation, and coalescence) and both the transport and production thresholds for charged particles and photons were set to 1 keV in the region of interest (the region around and within the scoring cavities; see also Section 2.C). In order to further enhance the transport precision for the simulations of the energy deposition in the small cavities, the multiple Coulomb scattering was suppressed in these regions by adding the MULSOPT card to the input file. Using this card, single scattering was activated and the minimum step length for multiple Coulomb scattering was increased by a factor of 10 000 to force the code to simulate the Coulomb scattering as detailed as possible.

Since the standard material definitions in fluka consider the natural isotopic composition of a given element (e.g., carbon consists of 98.9% ${}^{12}$C and 1.1% ${}^{13}$C), but in our material definitions (see Table 1), only the main isotopes are present (e.g., carbon consists of 100% ${}^{12}$C), the fluka material definitions were adapted.

The statistical uncertainties were estimated by calculating the standard deviation of the results from independent runs performed with different random seeds.^36^

### 2.E. Monte Carlo code 3: geant4/topas

Simulations were performed with the TOPAS code (“TOol for PArticle Simulation”)^42^ version 3.1.p1, a toolkit based on Geant4 (“GEometry ANd Tracking”)^43^ version geant4‐10‐03‐patch‐01. Since TOPAS is based on GEANT4, it uses the same physics models, processes, and interaction cross sections. Previous studies have extensively validated the code against experimental data.^42^, ^44^ The code is capable of transporting various kinds of particles including photons, electrons, positrons, neutrons, protons, and heavy ions.

In Geant4, electromagnetic (EM) interactions of the charged particles are grouped in the condensed history (CH) approach. While discrete collisions with an energy loss above a user‐defined threshold are simulated one by one, angular deflections of all soft collisions are grouped at the end of a given step using a given multiple scattering (MSC) theory.^45^ Since a real trajectory is not a straight line, the lateral displacement is considered at the end of the step.

For the simulations with TOPAS, we investigated both the physics lists ***g4em‐standard_opt3*** and ***g4em‐standard_opt4***. The physics list ***g4em‐standard_opt3*** makes use of the *G4UrbanMscModel* 46 for the multiple scattering of all charged particles. O’Brien et al.^47^ showed that when using the Urban scattering model, the Fano test is passed in clinical photon radiation fields within 0.1%. The physics list ***g4em‐standard_opt4*** makes use of the *WentzelVI* model^48^ as well as the Goudsmit–Saunderson model^49^, ^50^ for the multiple scattering: For electrons and positrons with energies below 100 MeV, the Goudsmit–Saunderson model is used. For electrons and positrons with an energy above 100 Mev and for protons with energies below 500 MeV, the WentzelVI model is used. It was shown that the WentzelVI model shows better agreement in the proton therapy range compared to the Urban model.^51^ Furthermore, Wulff et al.^16^ showed that the Fano test is passed in clinical proton beams within 0.1%–0.2% (depending on the beam geometry) when using the physics list ***g4em‐standard_opt4***.

The results obtained when using the physics list ***g4em‐standard_opt4*** for the photon simulations were unreasonable in terms of the $f_{Q}$ factor as described in Appendix A. For the proton simulations, the differences for the results between the physics lists ***g4em‐standard_opt3*** and ***g4em‐standard_opt4*** were small (<0.4% in terms of the $f_{Q}$ factor). Hence, we decided to only show the results obtained with the physics list ***g4em‐standard_opt3*** in the main text and show the results from the physics list ***g4em‐standard_opt4*** in Appendix A.

The length of a step in the CH is limited by tracking limits, such as geometric boundaries and physics‐related parameters. A parameter to control the step length is the parameter *dRoverR* which defines the maximum length of one single step in relation to the range of the particle. For the photon simulations, this parameter *dRoverR* was set to 0.003 following O’Brien et al.^47^ The fixed step size limiter *MaximumStepSize* was disabled (by setting it to 1000 m) also following O’Brien et al.^47^ For the proton simulations, the parameter *dRoverR* was set to 0.05 following a study by Wulff et al.^16^ and the *MaximumStepSize* was set to 1000 m, too. Further step limitations are combined in the *G4MscStepLimitType* which was set to *fUseDistanceToBoundary* for electrons and positrons in the used physics list.

While losing energy, the CH step length for each particle decreases until it is smaller than the *finalRange*, below which the particle is ranged out in a single straight step. The *finalRange* was set to 1 nm in the photon simulations and to 100 nm in the proton simulations.

For Compton scattering simulations, the *G4KleinNishinaModel* 53 was used. For ion ionization, the *G4IonParametrisedLossModel* based on the ICRU73^54^ ion stopping data was applied.

The simulation of nonelastic nuclear interactions was managed by the physics list ***g4h‐phy_QGSP_BIC_HP***. For inelastic nucleon–nucleus processes, the Binary Cascade model^55^ was used. To get inelastic cross sections, the *G4BGGNucleonInelasticXS* was taken for protons and neutrons.

For elastic scattering processes, the *G4ChipsElasticModel* was used for protons and neutrons from 0 to 100 TeV. *ChipsProtonElasticXS* provided the proton cross sections while *G4NeutronElasticXS* provided the neutron cross sections.

The default physics list ***g4ion-binarycascade*** was implemented so that the Binary Cascade model was also used for inelastic nuclear interactions of ions. The physics list ***g4decay*** was implemented in order to calculate the decay of particles like muons. Furthermore, the physics lists ***g4h-elastic_HP*** and ***g4stopping*** were used for high‐precission calculation of elastic processes of hadrons and to activate and provide the nuclear capture of negatively charged particles at rest.

The parameter controlling the production of secondaries is given in units of length in Geant4. The reason for this is that using a range instead of energy for the production thresholds is theoretically the more precise determination of the location for the energy release.^45^ The production cut for all particles was set to 0.5 mm in the whole geometry (which corresponds to ∼200 keV electrons in water), except in a region enveloping and including the scoring volume where the cut was set to 0.065 μm (which corresponds to ∼1 keV electrons in water). For the simulations of the air‐filled cavities, the cut in air was set to 47.2 μm, which corresponds to 990 eV electrons in air. The enveloping region was — equally to the setup in penh in Section 2.C set to be equal to the $R_{\mathrm{CSDA}}$ in water of 200 keV electrons, multiplied by a safety factor of 1.2.

The parameters explained in the text are also listed in Tables 3 and 4. No variance reduction techniques were used. The statistical uncertainties were estimated by combining the uncertainties from independent runs performed with different random seeds as described in Ref. [56].

**Table 3:** Production cuts and transport simulation parameters used in geant4/topas for the photon and proton simulations.

Region	Production cut in μm	Maximum stepsize in m	*dRoverR*		*finalRange* in nm	
			Photon‐sim.	Proton‐sim.	Photon‐sim.	Proton‐sim.
Scoring volume and envelope	0.065 for water and 47.2 for air	1000	0.003	0.05	1	100
Water phantom	500	1000	0.003	0.05	1	100

**Table 4:** Multiple scattering models used in geant4/topas for the photon and proton simulations

Radiation field	Multiple scattering model for e${}^{+}$/e${}^{-}$	Multiple scattering model for primaries
1.25 MeV photons	Urban model	/
150 MeV protons	Goudsmit–Saunderson (E ≤ 100 MeV) Wentzel VI (E > 100 MeV)	Wentzel VI (E ≤ 500 MeV)

To score the spectral fluence of primary photons and protons as well as the spectra of secondary electrons, we did not use the default scorer *fluence* provided by TOPAS. Instead, we took a self‐programmed scorer as described in the Appendix B.

### 2.F. Monte Carlo code 4: egsnrc

The egsnrc code is capable of transporting photons, electrons, and positrons and is widely used for dosimetric applications in medical physics. It was included in this study as a benchmark for the 1.25 MeV photon case; details of the transport models and physics behind the code can be found in Ref. [57]. It has been shown that the code is able to calculate the response of ionization chambers with an accuracy of 0.1% normalized to its own cross sections.^58^, ^59^ All simulations in this study were performed with the egsnrc version 2017, applying the user codes *egs_chamber* 60 for dose calculations and *cavity* 61 as well as *FLURZnrc* 62 for fluence calculations. Photons, electrons, and positrons were transported down to a kinetic energy of 1 keV (total energy of 512 keV for electrons/positrons). The energy thresholds for the production of secondary particles from electron interactions (*δ*‐electrons/bremsstrahlungs‐photons) was set to AE = 512 keV (*δ*‐electrons) and AP = 1 keV (bremsstrahlungs‐photons). Further transport parameters and applied cross sections are summarized in Table 5. Statistical uncertainties were estimated using the history‐by‐history method.^63^

**Table 5:** Transport simulation parameters used in the egsnrc simulations

Photon cross section	NIST
Brems cross section	KM
Brems angular sampling	KM
Electron Impact Ionization	ik
Rayleigh scattering	ON
Spin effects	ON
Bound Compton Scattering	ON
Radiative Compton corrections	ON
Atomic relaxations	ON
Pair angular sampling	KM
Triplet production	ON
PE angular sampling	ON
Photonuclear attenuation	ON
Photonuclear cross section	default
Boundary crossing algorithm	Exact
Skin depth for BCA	3
Electron‐step algorithm	egsnrc

## 3 Results

### 3.A. Absorbed dose in the water and air cavities

**Figure 2:**
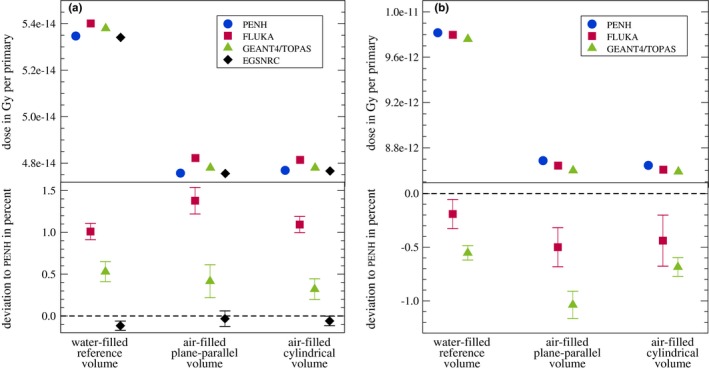
Absorbed dose in Gy per primary scored in each volume from Fig. 1 for all Monte Carlo codes. (a) 1.25 MeV photons, (b) 150 MeV protons. In the bottom graph, the deviations relative to penh are shown (see text for explanation). The statistical uncertainties of the absolute absorbed doses are smaller than the symbol size. The statistical uncertainties represented by bars in the bottom graphs correspond to one standard deviation. [Color figure can be viewed at http://www.wileyonlinelibrary.com/]

The results for the absorbed dose in the different geometries given in Fig. 1 for the Monte Carlo codes investigated are shown in Fig. 2; in panel (a) for the irradiation with 1.25 MeV photons and in panel (b) for the irradiation with 150 MeV protons. In the bottom graphs, the deviations relative to the penh results are shown to visualize the relative differences between the codes. Since no experimental data are available, we chose to investigate the deviations relative to one of the Monte Carlo codes used. Our choice fell on penh because, among the codes capable of transporting both electrons and protons, it was the one that reached the smallest statistical uncertainty within a reasonable calculation time. However, this normalization is still arbitrary and does not claim that penh gave the most accurate results.

For the irradiation with photons, the absorbed doses in the water‐filled reference volume are for all Monte Carlo codes ∼12% larger than the absorbed doses in the air‐filled volumes.

The absorbed doses for the Monte Carlo codes penh and egsnrc agree within one standard deviation for the air‐filled volumes and within two standard deviations for the water‐filled volume. The largest deviation between these two codes is 0.1%. The absorbed doses calculated with fluka are up to 1.4% larger compared to penh, those calculated with geant4/topas are up to 0.5% larger.

For the irradiation with protons, the absorbed doses in the water‐filled reference volume are for all Monte Carlo codes ∼13% larger than the absorbed doses in the air‐filled volumes.

The absorbed doses calculated with fluka are up to 0.5% smaller compared to penh, those obtained with geant4/topas are up to 1.0% smaller.

### 3.B. $f_{Q_{0}}$ factors, $f_{Q}$ factors, and $f_{Q}/f_{Q_{0}}$ ratios

**Figure 3:**
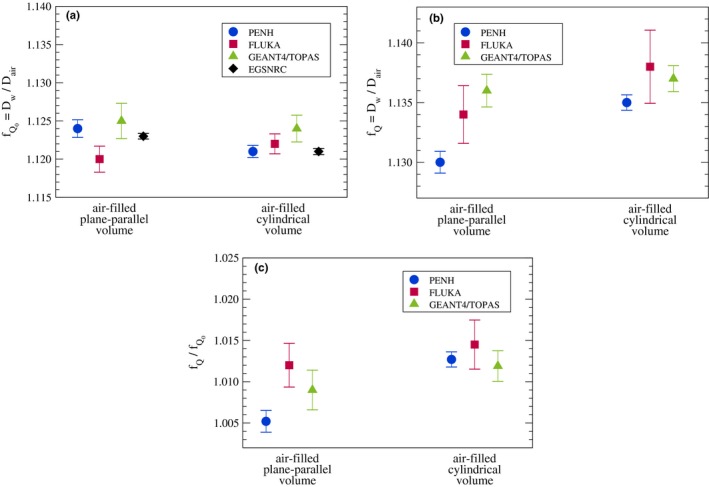
Results for $f_{Q_{0}}$ and $f_{Q}$ factors: (a) 1.25 MeV photons and (b) 150 MeV protons. (c) The $f_{Q}/f_{Q_{0}}$ ratios for 150 MeV protons. The statistical uncertainties represented by bars correspond to one standard deviation. [Color figure can be viewed at http://www.wileyonlinelibrary.com/]

In Fig. 3, the $f_{Q_{0}}$ and $f_{Q}$ factors calculated with Eq. (1) as well as the $f_{Q}/f_{Q_{0}}$ ratios are summarized.

The $f_{Q_{0}}$ factors for the irradiation with 1.25 MeV photons shown in panel (a) are in the range of ∼1.120–1.125. The results for all codes agree within two standard deviations or better. The largest deviation is between fluka and geant4/topas for the plane‐parallel cavity with 0.4% .

The $f_{Q}$ factors for the irradiation with 150 MeV protons are shown in panel (b). For both volumes, the $f_{Q}$ factors are in the range of 1.130–1.138. Except for the plane‐parallel volume and the codes penh and geant4/topas, the results for all codes coincide within two standard deviations. The largest deviation is that between penh and geant4/topas for the plane‐parallel volume with 0.5%.

In panel (c), the $f_{Q}/f_{Q_{0}}$ ratios are shown for 150 MeV protons and both air‐filled volumes. The $f_{Q}/f_{Q_{0}}$ ratios are in the range of 1.005–1.015. For both volumes, the results for all codes coincide within two standard deviations or better. The largest difference can be seen for the plane‐parallel volume and the codes penh and fluka with a deviation of 0.7%.

The ratios $f_{Q}/f_{Q_{0}}$ from Fig. 3 were calculated for each of the codes penh, fluka and geant4/topas using the $f_{Q_{0}}$ and $f_{Q}$ factors both calculated with the same code. While $f_{Q}$ has to be calculated with a code capable of transporting protons if *Q* is a proton radiation field, the $f_{Q_{0}}$ factor could theoretically be calculated using another code such as egsnrc being a commonly used code for the calculations of photons. In Table 6, the $f_{Q}/f_{Q_{0}}$ ratios for the codes penh, fluka, and geant4/topas are listed for both air‐filled volumes once determined using the $f_{Q_{0}}$ and $f_{Q}$ factors both calculated with the same code vs the results when using $f_{Q_{0}}$ calculated with egsnrc. The differences are smaller than or equal to their statistical uncertainties; hence, we cannot conclude any differences.

**Table 6:** $f_{Q}/f_{Q_{0}}$ ratios for the Monte Carlo codes penh, fluka, and geant4/topas using the $f_{Q_{0}}$ factors calculated with the same Monte Carlo code and using $f_{Q_{0}}$ determined with egsnrc. The given statistical uncertainties are one standard deviation.

Monte Carlo code	Volume	ratio $f_{Q}$/$f_{Q_{0}}$		Difference in %
		$f_{Q_{0}}$ from same code	$f_{Q_{0}}$ from egsnrc	
penh	Plane‐parallel	1.005 ± 0.001	1.006 ± 0.001	−0.09 ± 0.16
	Cylindrical	1.013 ± 0.001	1.013 ± 0.001	−0.06 ± 0.11
fluka	Plane‐parallel	1.012 ± 0.003	1.009 ± 0.002	0.3 ± 0.3
	Cylindrical	1.015 ± 0.003	1.016 ± 0.003	−0.1 ± 0.4
geant4/topas	Plane‐parallel	1.009 ± 0.002	1.011 ± 0.001	−0.2 ± 0.3
	Cylindrical	1.012 ± 0.002	1.015 ± 0.001	−0.3 ± 0.2

### 3.C. Spectral fluences in water

**Figure 4:**
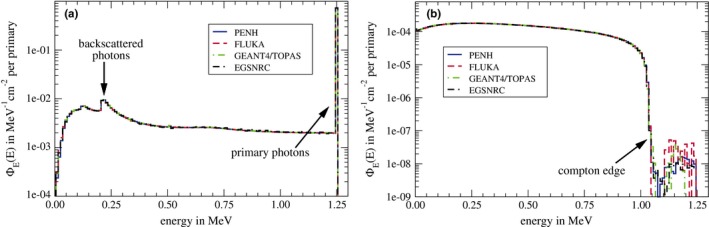
Spectral fluence $\Phi_{E}\left(E\right)$ in water of photons (a) and electrons (b) in the 1.25 MeV photon beam at a depth of 5 cm. [Color figure can be viewed at http://www.wileyonlinelibrary.com/]

Figure 4 shows the spectral fluences in water of the photons (a) and the secondary electrons (b) within the water‐filled reference volume for the irradiation with 1.25 MeV photons. The peak of the primary photons at an energy of 1.25 MeV can clearly be seen. The spectrum of the Compton‐scattered photons with the peak of the backscattered photons at an energy of ∼0.2 MeV is also clearly visible. Accordingly, the Compton edge can be seen in the spectral fluence of the secondary electrons at an energy of ∼1.05 MeV. The broad Compton spectrum is clearly visible, too. The spectra for both the photons and electrons agree almost perfectly between all Monte Carlo codes.

**Figure 5:**
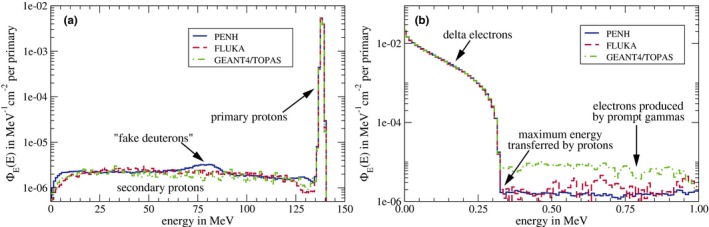
Spectral fluence $\Phi_{E}\left(E\right)$ in water of protons (a) and electrons (b) in the 150 MeV proton beam at a depth of 2 cm. [Color figure can be viewed at http://www.wileyonlinelibrary.com/]

The spectral fluence in water of protons in the 150 MeV proton beam at the cavity position is shown in Fig. 5. The peak of the primary protons lies at an energy of ∼139 MeV (corresponding to 150 MeV minus the energy loss in the first 2 cm of water). The spectrum of the secondary protons ranges over the whole spectrum up to the primary protons. Differences between the individual codes can be observed for very low energies up to ∼20 MeV and higher energies between 125 and 135 MeV. The spectral fluence of the secondary protons scored with penh develops a small peak at an energy of ∼80 MeV. This might be due to the fact that in penh fragments heavier than protons (like deuterons) are simulated as secondary protons and the energy of these “protons” is adjusted to match the range of the real fragments.^30^ In this case, these fragments are deuterons that are simulated as 80 MeV protons which would have a range in water of about 5 cm.

The spectral fluence in water of the delta electrons in the 150 MeV proton beam can be seen in Fig. 5(b). As expected, the fluence is dominated by low‐energy electrons. The maximum energy transferred to electrons in ionization processes by 139 MeV protons [compare energy of primary protons in Fig. 5(a)] is ∼0.3 MeV.^64^ Electrons with higher energies are not produced by protons but by prompt gamma photons from nuclear reactions. The spectral fluence of these high‐energy electrons is considerably larger for geant4/topas than for penh and fluka.

For all codes, there are no relevant differences between the individual spectra except for the secondary protons and electrons produced by prompt gamma photons as pointed out.

### 3.D. Electronic and nuclear buildup, ranges, and stopping powers

**Figure 6:**
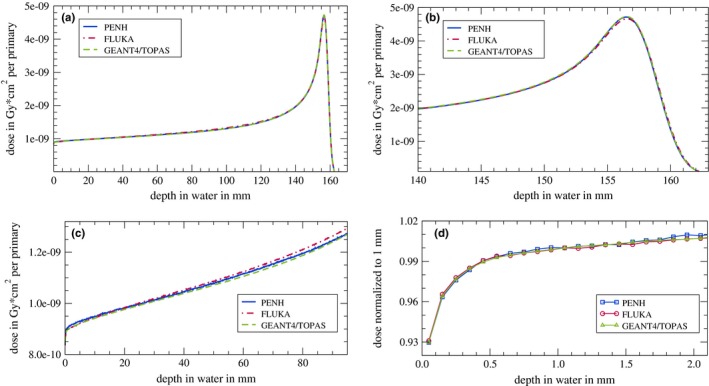
(a) Integrated depth–dose curve of 150 MeV protons in water (dose integrated over an area of 100 cm${}^{2}$). (b) Zoom to the Bragg peak. (c) Zoom to the first 90 mm. (d) Zoom to the first 2 mm while the dose values are normalized to the dose at a depth of 1 mm. The single data points in (d) are connected with lines to guide the eye. No statistical uncertainties are indicated since one standard deviation is smaller than the line width [panels (a)–(c)] or the symbol size [panel (d)]. [Color figure can be viewed at http://www.wileyonlinelibrary.com/]

Figure 6(a) shows the integrated depth–dose curve of 150 MeV protons in water. Among all codes, the dose curves are in a good agreement. In panel (b), a zoom to the Bragg peak is shown. As can be seen, the dose at the peak calculated with fluka is about ∼1.5% smaller compared to the doses calculated with penh and geant4/topas. Furthermore, the ranges of all codes agree within ∼0.1 mm which is the resolution of the scored dose curves. In panel (c), a zoom to the first 95 mm is shown. In this region, the secondary proton buildup takes place. Small discrepancies between the codes can be seen at small depths of ∼5 mm (1.3% difference in dose) and at greater depth of ∼70 mm and ∼90 mm (1.5% difference in dose). These differences are statistically significant. The best agreement between the codes is at depths of 20–40 mm supporting the IAEA reference depth for monoenergetic protons of 3 g cm.${}^{-2}$ Note that these dose curves were produced with a pencil beam and the dose was laterally integrated. Hence, the absolute doses differ from the ones presented in Fig. 2(b).

In Fig. 6(d), the first 2 mm of the Bragg curve is shown while the dose values are normalized to the dose value at a depth of 1 mm. As already mentioned, this region provides an excellent test of the electromagnetic interaction models implemented in the different Monte Carlo codes. Within the first millimeters of the depth–dose curve, the buildup is dominated by the creation of delta electrons with energies up to 0.33 MeV.^64^ The range of these electrons is about 1 mm. Accordingly, an equilibrium is reached and the electron buildup is completed at this depth [see Fig. 6(d)]. The electron buildup is reproduced by all three Monte Carlo codes identically, indicating that the underlying electromagnetic interaction models are similar in these codes [also compare Fig. 5(b)]. The completion of the electronic buildup at a depth of 1 mm is the reason why the depth–dose curves are normalized to the dose at this depth.

**Figure 7:**
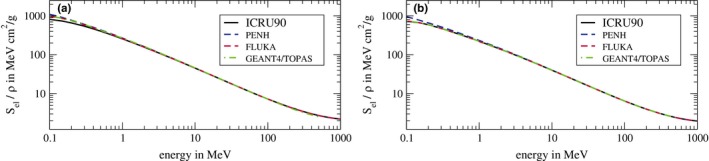
Electronic mass‐stopping powers of (a) water and (b) air for protons. The data labeled with *ICRU90* are taken from Ref. [17]. [Color figure can be viewed at http://www.wileyonlinelibrary.com/]

To ensure that the stopping powers are the same for the different Monte Carlo codes for the given mean ionization potentials of water $I_{w}$ = 78 eV and air $I_{\mathrm{air}}$ = 85.7 eV, we compared the stopping powers of water and air for protons and electrons. In Fig. 7, the electronic mass‐stopping powers of (a) water and (b) air for protons for the Monte Carlo codes penh, fluka, and geant4/topas are shown. As reference, the ICRU90 data^17^ are shown. For energies between 0.3 MeV and 1000 MeV, there are no significant differences between the codes and the ICRU90 data. Only for energies below 0.3 MeV, differences can be seen. The stopping powers of water and air for electrons agreed almost perfectly among the Monte Carlo codes and with the reference data from the ICRU90 in the energy range from 10 keV to 900 MeV (not shown).

## 4 Discussion

Simulations of the absorbed dose in simple water‐ and air‐filled geometries and the calculation of $f_{Q}$ factors as well as $f_{Q}/f_{Q_{0}}$ ratios were carried out with different Monte Carlo codes for different beam qualities and particle types. Although the absorbed dose to medium per primary particle does not always agree for all Monte Carlo codes, these differences tend to cancel out in the $f_{Q}$ factors and the $f_{Q}/f_{Q_{0}}$ ratios. The $f_{Q}/f_{Q_{0}}$ ratios agree within 0.7% for protons for the Monte Carlo codes penh, fluka, and geant4/topas. Since these ratios are the only part of $k_{Q,Q_{0}}$ factors that can be determined with the Monte Carlo method and since it was shown, that penh can be used to calculate $k_{Q,Q_{0}}$ factors in clinical proton beams,^15^ we conclude that fluka and geant4/topas can also be used to calculate $k_{Q,Q_{0}}$ factors in clinical proton beams.

When mixing the codes in a way that the $f_{Q}$ factor is taken from a code capable of transporting protons and $f_{Q_{0}}$ is derived using egsnrc, the differences in the $f_{Q}/f_{Q_{0}}$ ratios are not significant compared to the case when both $f_{Q}$ and $f_{Q_{0}}$ are calculated within the same code. Although no significant differences were seen, it is commonly advised to always use the same code to calculate the $f_{Q}$ as well as the $f_{Q_{0}}$ factor to avoid a mixing of physical models and to enhance the chance that code‐internal systematic uncertainties are canceled out in the $D_{w}/D_{\mathrm{air}}$ ratios, that is, in the $f_{Q}$ factors, as well as the $f_{Q}/f_{Q_{0}}$ ratios.

The $f_{Q_{0}}$ factors for 1.25 MeV photons for the air‐filled volumes are in the range of 1.120–1.125 and the $f_{Q}$ factors for 150 MeV protons for the air‐filled volumes are in the range of 1.130–1.138. These factors correspond approximately to the Spencer–Attix water to air mass stopping power ratio $s_{w,\mathrm{air}}$. For a ${}^{60}$Co beam, this mass stopping power ratio is ∼1.127^65^, ^66^, and for a 150 MeV proton beam, it is 1.130.^67^ The $f_{Q_{0}}$ and $f_{Q}$ factors calculated in this study agree with these values within a few permille. Differences are due to the fact that the stopping power ratio is defined on the fluence in water, whereas in this work, we scored the fluence in air.

In order to try to explain differences in the particle transport and the physics models used in the Monte Carlo codes, we presented spectral fluences in water of the primary particles and secondary electrons. In spite of possible differences due to the use of different approaches and models, we found remarkable agreements between the codes: For the photons and secondary electrons in the 1.25 MeV photon field, the spectra agree almost perfectly. For the spectral fluence of protons and electrons in the 150 MeV proton field, the spectra also agree reasonably well, except for the secondary protons and high‐energy electrons produced by prompt gamma photons. For the secondary protons, we observed differences between the spectra generated with the different Monte Carlo codes for energies between 125 and 135 MeV. The production of a secondary proton from an ${}^{16}$O nucleus requires an energy of ∼12 MeV,^68^; therefore, the gap between the primary and secondary protons visible in the spectra generated with TOPAS and fluka is reasonable. However, this gap cannot be observed in the spectrum generated with penh. Anyway, this difference in the spectral fluence of secondary protons has only a very small effect on the absorbed dose $D_{w}$ at a water depth of 2 cm (where the spectral fluence and the absorbed dose were calculated), since the fluence of secondary protons is >3 orders of magnitudes smaller than the fluence of primary protons.

The spectral fluence of high‐energy electrons produced by prompt gamma photons is considerably larger for geant4/topas than for penh and fluka. It was shown by Robert et al. that in Geant4, many more prompt gamma photons are produced compared to fluka,^40^ which might be the explanation for the differences observed in this work. However, for dosimetry purposes, these differences in the prompt gamma photon production are negligible since the fluence of electrons generated by prompt gamma photons is >2 orders of magnitudes smaller than the fluence of the delta electrons.

On the one hand, the agreement in the spectra of secondary electrons shows that the different electromagnetic interaction models yield similar results. On the other hand, the differences in the spectra of secondary protons show that the more complex nuclear interaction models tend to slight differences. Accordingly, discrepancies in the proton depth–dose curves as shown in Fig. 6 due to the different predictions of the secondary proton production were observed. However, the impact of these differences at small depths is so low that the $f_{Q}/f_{Q_{0}}$ ratios at a water depth of 2 cm calculated with the different codes agree within 0.7%. Pfuhl et al.^27^ have compared dose profiles similar to those shown in Fig. 6(d) with measured dose profiles. fluka could reproduce both the electronic and the nuclear buildup quite well, with a maximum underestimation of ∼1% at large depths. In another study by Yang et al.^69^, depth–dose curves were calculated with both fluka and Geant4 and compared to measurements. Yang et al. concluded that fluka and Geant4 are “capable of performing dose calculations for therapeutic scanning proton beams with proper physics settings.”^69^ Furthermore, Monte Carlo codes like, for example, fluka are already used to generate basic data for treatment‐planning systems and are hence intensively validated against experimental dose measurements.^70^, ^71^

We also showed that the ranges of protons in water agree within ∼0.1 mm between the codes [see Fig 6(b)]. Since the range is connected with the stopping power of water, we investigated the stopping powers of water for protons and electrons and found good agreements between the codes and the ICRU90 stopping powers. The only differences for protons were found for energies below 0.3 MeV. Since the range in water of protons with an energy of 0.3 MeV is only ∼5 μm, these differences in the stopping powers have no visible effect on the range that we calculated with a resolution of 0.1 mm.

Finally, a last consideration regarding uncertainties in Monte Carlo calculations: while type A uncertainties are easy to calculate and can be reduced by increasing the number of primary histories, type B uncertainties (due to uncertainties in the cross sections, nuclear models, particle transport, geometry, etc.) are more difficult to estimate. The results of this work could be used to estimate (an upper limit to) the type B uncertainty in the $f_{Q}/f_{Q_{0}}$ ratios calculated with penh, fluka, and geant4/topas, due to the uncertainty in cross‐section data, nuclear models, and particle transport. Figure 3(c) shows that the differences in $f_{Q}/f_{Q_{0}}$ ratios are well within three (type A) standard uncertainties of the data points calculated with fluka. Thus, it could be concluded that an upper limit to the type B uncertainty in the $f_{Q}/f_{Q_{0}}$ ratios (due to the uncertainty in cross‐section data, nuclear models, and particle transport) is the type A uncertainty in the $f_{Q}/f_{Q_{0}}$ ratios calculated with fluka. That is, $u_{B}$ ≤ 0.3%.

## 5 Conclusion

The Monte Carlo codes penh , fluka, geant4/topas, and egsnrc were investigated and used to calculate the absorbed dose in a simple water‐filled reference volume and two air‐filled volumes representing simplified ionization chambers for the irradiation with 1.25 MeV photons representing a ${}^{60}$Co beam and with 150 MeV protons — egsnrc was used only for the photon beam calculations. The absorbed doses to medium (per primary particle) agreed within 1.4% or better for the photon field among all codes. For the irradiation with 150 MeV protons, the absorbed doses to medium (per primary particle) agreed within 1.0% or better among the three codes capable of transporting protons (penh, fluka, and TOPAS/Geant4). It was shown that by choosing appropriate transport settings which were reported in detail, the results for the $f_{Q}/f_{Q_{0}}$ ratios agreed within 0.7%. In other words, penh, fluka, and TOPAS/Geant4 are all suitable to calculate $f_{Q_{0}}$, $f_{Q}$, and $k_{Q,Q_{0}}$ factors in proton beams.

## Figures and Tables

**Figure 8:**
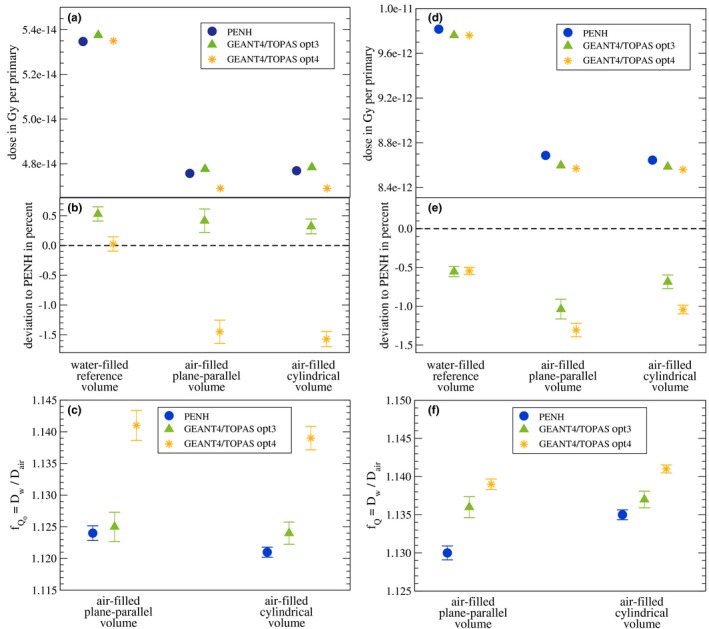
Absorbed dose in Gy per primary scored in each volume from Fig. 1 for the 1.25 MeV photons (a) and 150 MeV protons (d) calculated with penh and geant4/topas using the physics lists *g4em‐standard_opt3* and *g4em‐standard_opt4*. In (b) and (e), the deviations relative to penh are shown. The statistical uncertainties of the absolute absorbed doses are smaller than the symbol size. The statistical uncertainties represented by bars in panel (b) and (e) correspond to one standard deviation. (c) The results for $f_{Q_{0}}$ factors for 1.25 MeV photons; (f) the results for $f_{Q}$ factors for 150 MeV protons. The statistical uncertainties represented by bars correspond to one standard deviation. [Color figure can be viewed at http://www.wileyonlinelibrary.com/]

**Figure 9:**
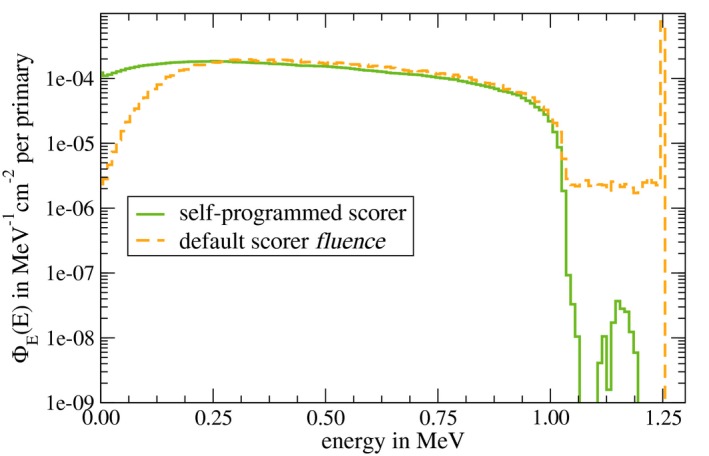
Energy‐dependent fluence $\Phi_{E}\left(E\right)$ in water of secondary electrons binned by energy in a 1.25 MeV photon beam scored in TOPAS with the self‐programmed scorer (fluence binned by energy the particle had when generating the scoring hit; corresponding to the spectral fluence) and the default scorer *fluence* implemented in TOPAS (fluence binned by the energy the particle had when entering the scoring volume). [Color figure can be viewed at http://www.wileyonlinelibrary.com/]

## Appendix A Physics list in topas for photon and proton fields

As described in Section 2.C, we used the physics list *g4em‐standard_opt3* for both the photon and proton simulations for the Monte Carlo code geant4/topas. We also tested the physics list *g4em‐standard_opt4* for both the photon and proton simulations. In Fig. 8, the absorbed doses for the three cavities calculated with penh and geant4/topas for the two physics lists as well as the deviations relative to penh are shown on the left side for photons [panels (a) and (b)]. The absorbed dose in the water‐filled reference volume is ∼0.5% smaller and the absorbed dose in the air‐filled cavities is ∼2% smaller when replacing the physics list *g4em‐standard_opt3* by *g4em‐standard_opt4*. Hence, the deviations relative to penh are up to −1.5% instead of 0.5%. In Fig. 8 in panel (c), the resulting $f_{Q_{0}}$ factors for photons are shown: when using the physics list *g4em‐standard_opt4*, the $f_{Q_{0}}$ factor is ∼1.141 instead of ∼1.125 and hence 1.4% greater and does not agree with the Spencer–Attix water to air mass stopping power ratio $s_{w,\mathrm{air}}$ anymore.

In panels (d) and (e), the absorbed doses for the three cavities calculated with penh and geant4/topas for the two physics lists as well as the deviations relative to penh are shown for protons. The difference in the absorbed dose is <0.1% for the water‐filled reference volume and <0.36% for the air‐filled cavities when replacing the physics list *g4em‐standard_opt3* by *g4em‐standard_opt4*. For the resulting $f_{Q}$ factors, the differences are smaller than 0.4% for the different physics lists. The maximum deviation between penh and geant4/topas rises from 0.5% for the physics list *g4em‐standard_opt3* to 0.8% for the physics list *g4em‐standard_opt4*.

Since the multiple scattering models used in the physics lists *g4em‐standard_opt3* and *g4em‐standard_opt4* are not the only differences we cannot clarify whether these scattering models are responsible for the different results.

In conclusion: for the proton simulations, both the physics lists *g4em‐standard_opt3* and *g4em‐standard_opt4* can be used whereas for the photon simulations, the physics list *g4em‐standard_opt4* does not lead to reasonable results. Additionally, one has to keep in mind that the physics lists provided by Geant4 may vary with the versions of the code. Hence, the physics lists used in this work may lead to different results in the future when changed in new versions of Geant4.

## Appendix B Scoring the spectral fluence in topas

As mentioned in Section 2.C, we used a self‐programmed scorer in TOPAS to score the spectral fluence in the water cavity. The reason is that the scorer *fluence* provided by TOPAS is not designed to score a **spectral** fluence. Although the fluence can be binned by energy, this energy binning is not based on the particle’s energy when the scoring hit is generated. The scorer *fluence* bins the fluence of the particle to the energy the particle had when the particle was incident on the scoring volume (i.e., when entering the scoring volume). However, this so‐scored energy‐binned fluence does not correspond to the spectral fluence of a particle. In the case that a particle is produced within the scoring volume, the difference between the result of the energy‐binned scorer *fluence* and the spectral fluence becomes clear: The scorer *fluence* provided by TOPAS connects the fluence of this particle created within the scoring volume to the energy its predecessor had when entering the scoring volume and not to the energy of the scored particle it had when the hit was generated — as it is the case when scoring the spectral fluence.

In Fig. 9, the fluences of electrons at 5 cm water depth in a 1.25 MeV photon beam are shown: In the first scenario, the fluence is binned by the energy the particle had when entering the scoring volume (as it is done in the scorer *fluence* provided by TOPAS). In the second scenario, the fluence is binned by the energy the particle had when generating the scoring hit (this corresponds to the spectral fluence and is the scoring method used in this study).

As can be seen, the fluence scored with the scorer *fluence* provided by TOPAS shows electrons with an energy of 1.25 MeV. These electrons were produced within the scoring volume, and hence, TOPAS bins the fluence of these electrons by the energy their predecessor (in this case a 1.25 MeV photon) had when entering the scoring volume. In contrast, the scorer used in this study for the TOPAS simulations does not show this peak since the fluence is binned by the energy of the particle itself that was created in the scoring volume. Correspondingly, differences in the fluence of low‐energy electrons can be seen: Since the scorer *fluence* provided by TOPAS bins the fluence of low‐energy electrons that are created within the scoring volume by the energy of their predecessors, the fluence of low‐energy electrons is smaller compared to the spectral fluence scored with the self‐programmed scorer which bins the fluence by the energy of the electron itself that was produced in the scoring volume. The spectral fluence scored with the self‐programmed scorer was already shown in Fig. 4 and proven to agree with the results from the Monte Carlo codes penh, fluka, and egsnrc.

To avoid confusion when using the scorer *fluence* provided by TOPAS, several scoring options are now included in the new TOPAS version 3.2.
